# Evidence for Altered Phosphoinositide Signaling-Associated Molecules in the Postmortem Prefrontal Cortex of Patients with Schizophrenia

**DOI:** 10.3390/ijms22158280

**Published:** 2021-07-31

**Authors:** Yasuto Kunii, Junya Matsumoto, Ryuta Izumi, Atsuko Nagaoka, Mizuki Hino, Risa Shishido, Makoto Sainouchi, Hiroyasu Akatsu, Yoshio Hashizume, Akiyoshi Kakita, Hirooki Yabe

**Affiliations:** 1 Department of Neuropsychiatry, School of Medicine, Fukushima Medical University, Fukushima 960-1295, Japan; junya.matsumoto@outlook.com (J.M.); r.123.izumi@gmail.com (R.I.); atsuko72n@gmail.com (A.N.); mhino@med.tohoku.ac.jp (M.H.); sisidokenshin42@gmail.com (R.S.); hyabe@fmu.ac.jp (H.Y.); 2 Department of Disaster Psychiatry, International Research Institute of Disaster Science, Tohoku University, Sendai 980-8573, Japan; 3 Department of Pathology, Brain Research Institute, Niigata University, Niigata 951-8585, Japan; m.sainouchi@gmail.com (M.S.); kakita@bri.niigata-u.ac.jp (A.K.); 4 Department of Community-Based Medical Education, Nagoya City University Graduate School of Medical Science, Aichi 467-8601, Japan; akatu@med.nagoya-cu.ac.jp; 5 Department of Community-Based Medicine, Nagoya City University Graduate School of Medical Science, Aichi 467-8601, Japan; 6 Choju Medical Institute, Fukushimura Hospital, Aichi 441-8124, Japan; yhashi@chojuken.net

**Keywords:** phosphoinositides, phosphatidylinositol 4-kinase alpha, protein kinase B, schizophrenia, postmortem brain, prefrontal cortex, multiplex immunoassay

## Abstract

Phosphoinositides (PIs) play important roles in the structure and function of the brain. Associations between PIs and the pathophysiology of schizophrenia have been studied. However, the significance of the PI metabolic pathway in the pathology of schizophrenia is unknown. We examined the expression of PI signaling-associated proteins in the postmortem brain of schizophrenia patients. Protein expression levels of phosphatidylinositol 4-phosphate 5-kinase type-1 gamma (PIP5K1C), phosphatidylinositol 4-kinase alpha (PIK4CA, also known as PIK4A), phosphatase and tensin homolog deleted from chromosome 10 (PTEN), protein kinase B (Akt), and glycogen synthase kinase 3β (GSK3β) were measured using enzyme-linked immunosorbent assays and multiplex fluorescent bead-based immunoassays of the prefrontal cortex (PFC) of postmortem samples from 23 schizophrenia patients and 47 normal controls. We also examined the association between PIK4CA expression and its genetic variants in the same brain samples. PIK4CA expression was lower, whereas Akt expression was higher, in the PFC of schizophrenia patients than in that of controls; PIP5K1C, PTEN, and GSK3β expression was not different. No single-nucleotide polymorphism significantly affected protein expression. We identified molecules involved in the pathology of schizophrenia via this lipid metabolic pathway. These results suggest that PIK4CA is involved in the mechanism underlying the pathogenesis of schizophrenia and is a potential novel therapeutic target.

## 1. Introduction

It is crucial to overcome schizophrenia because of its high prevalence, poor social prognosis, and considerable socio-economic cost [1]. However, as biological diagnostic indicators of schizophrenia have not been determined, the condition is still diagnosed symptomatically in clinical practice. Moreover, the treatment methods for schizophrenia are limited, and the outcomes of treatment, including improved social functioning, are not sufficient. Therefore, it is essential to elucidate the biological pathology of schizophrenia and develop new therapeutic methods.

**Figure 1:**
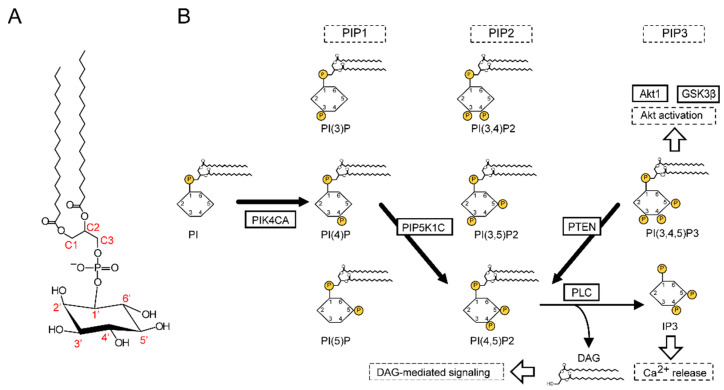
(**A**). Structure of phosphatidylinositol (PI). PI is composed of fatty acid residues attached to the C1 and C2 positions of the glycerol backbone, and inositol is attached via phosphate at the C3 position. The hydroxyl groups at positions 3′, 4′, and 5′ of the inositol head can undergo additional phosphorylation. As there are more than 30 different combinations of fatty acid residues and 8 different phosphorylation states are possible, there are more than 240 molecular species of PI in vivo. (**B**). Schematic diagram of phosphoinositide signaling and associated proteins analyzed in this study.

The pathology of schizophrenia involves changes in the brain, and total lipids make up half of the brains dry weight. Moreover, approximately 60% of total lipids are phospholipids [2,3,4], which are amphipathic molecules that form a lipid bilayer and are the main component of biological membranes. Phospholipids are classified into glycerophospholipids and sphingophospholipids based on their backbone. Among the glycerophospholipids, some are abundant in the cell membrane, such as phosphatidylcholine, phosphatidylethanolamine, phosphatidylserine, and phosphatidylinositol (PI) [5,6] (Figure 1A). Phosphoinositides (PIs), a collective term for PI and its phosphorylated derivatives, are involved in intracellular signal transduction as substrates for second messenger production (Figure 1A,B). Additionally, they localize target proteins to biological membranes by binding to their specific binding motifs. Structurally, fatty acid residues are bound to the C1 and C2 positions of the glycerol backbone of PI, and inositol, which can be phosphorylated at positions 3, 4, and 5 to produce PIs, is bound via the phosphate at the C3 position. More than 240 PIs have been identified in vivo as more than 30 combinations of fatty acid residues and eight phosphorylation states are possible [7,8].

**Figure 2:**
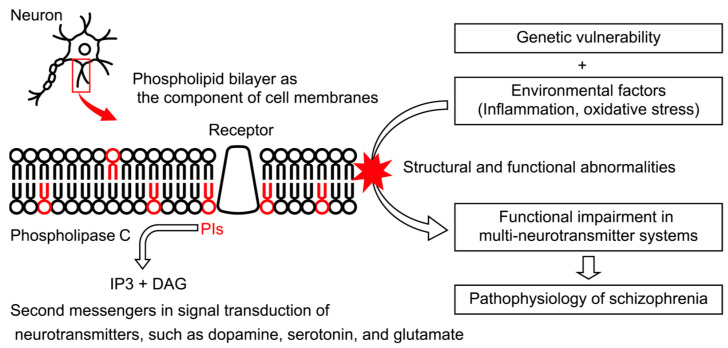
Schematic diagram of the association between phosphoinositides (PIs) and pathophysiology of schizophrenia. PIs are one of the components that make up the cell membrane of neurons. PIs also function as second messengers for neurotransmitter signaling. The signaling of dopamine, serotonin, and glutamate, which is associated with schizophrenia, converges with the activation of phospholipase C (PLC) and the production of inositol trisphosphate (IP3) and diacylglycerol (DAG) as second messengers from PIs. In schizophrenia, genetic vulnerability and environmental factors may lead to functional impairment in multi-neurotransmitter systems through structural and functional dysfunction of PIs.

Phospholipids play a crucial role in the structure and function of the brain [6]. They are degraded by several classes of phospholipases and act as second messengers in signaling pathways in neural and glial cells [9]. Therefore, adequate phospholipid production is essential for normal brain function, and alterations may be associated with the pathophysiology of schizophrenia [5] (Figure 2). Studies have focused on the relationship between schizophrenia and lipids since the 1970s, when researchers suggested that the prostaglandins synthesized from phospholipids are associated with schizophrenia [10,11]. Multiple studies have shown decreased levels of polyunsaturated fatty acids in the cell membrane of red blood cells from patients with schizophrenia, and this discovery has been confirmed by meta-analyses [12,13]. However, because a Cochrane review [14] indicated that the clinical effects of polyunsaturated fatty acid administration for schizophrenia have been inconsistent among studies, it was suggested that alterations in fatty acid levels are not the main contributor to the pathophysiology of schizophrenia. Instead, to elucidate the cause of schizophrenia, researchers need to focus on phospholipids, which are the main source of lipid signaling-associated fatty acids in the brain tissue [15].

Indeed, many studies of lipid analysis using postmortem brain tissues from patients with schizophrenia have been conducted by using various analytical technologies such as, magnetic resonance spectroscopy [16,17], high performance liquid chromatography [18,19], and gas chromatography [20,21]. However, most of these studies could not measure PIs with specific fatty acid combinations. In our previous studies using liquid chromatography-electrospray ionization mass/mass spectrometry and imaging mass spectrometry [22,23], we showed that although the levels of most phospholipid molecular species in the postmortem prefrontal cortex (PFC) were not different between subjects with schizophrenia and controls, the levels of some species were lower in the PFC from patients with schizophrenia than in that from controls.

However, the metabolic pathway of PIs is complex and, currently, its association with the pathophysiology of schizophrenia is unknown. In this study, we investigated the expression of proteins involved in the phosphorylation/dephosphorylation of PIs and the subsequent signal transduction pathway in the postmortem brain of patients with schizophrenia. Specifically, in the PFC, we analyzed the protein expression of phosphatidylinositol 4-phosphate 5-kinase type-1 gamma (PIP5K1C), phosphatidylinositol 4-kinase alpha (PIK4CA), and phosphatase and tensin homolog deleted from chromosome 10 (PTEN) as upstream, midstream, and downstream enzymes of the metabolic pathway of PIs. PIP5K1C belongs to a family with three members. Among them is the PIP5K1C that was studied here, which is predominantly expressed in the brain [24,25]. The PIP5K1C (PIP5Kγ) has three splicing variants, PIP5Kγ635, PIP5γ661, and PIP5Kγ687, but in this study we measured total protein levels of PIP5Kγ including all three variants without distinguishing between splicing variants. We also measured the protein expression of protein kinase B (Akt1) and its downstream factor, glycogen synthase kinase 3β (GSK3β), because phosphatidylinositol (3,4,5)-trisphosphate (PI(3,4,5)P3), one of the fully phosphorylated PIs, activates Akt by recruiting it to the plasma membrane, thereby allowing its phosphorylation by 3-phosphoinositide-dependent protein kinase 1 and mechanistic target of rapamycin complex 2 on Thr308 and Ser473, which are important for its catalytic activity [26]. Moreover, we examined the relationship between the expression levels and gene polymorphisms (single-nucleotide polymorphisms [SNPs]) of *PIP5K1C*, *PI4KA*, *PTEN*, and *GSK3B*.

## 2. Results

### 2.1. Expression of Phospholipid Signaling-Associated Molecules in PFC of Patients with Schizophrenia and Controls

**Figure 3:**
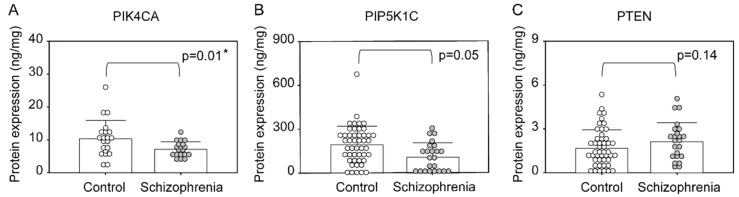
Expression levels of phosphoinositide signaling-associated proteins in the prefrontal cortex of patients with schizophrenia and controls. (**A**): PIK4CA, (**B**): PIP5K1C, (**C**): PTEN. Means ± SD are shown as bars and whiskers. * *p* < 0.05.

**Figure 4:**
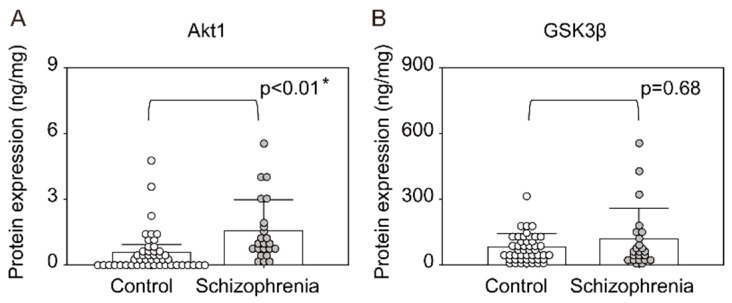
Expression levels of proteins downstream of phosphoinositide signaling pathway in the prefrontal cortex of patients with schizophrenia and controls. (**A**): Akt1, (**B**): GSK3β. Means ± SD are shown as bars and whiskers. * *p* < 0.05.

The PIK4CA level in the PFC was significantly lower in patients with schizophrenia than in controls (*p* = 0.01, Figure 3A). The levels of PIP5K1C and PTEN in the PFC did not differ significantly between the groups (Figure 3B,C). The level of Akt in PFC was significantly higher in patients with schizophrenia than in controls (*p* < 0.01, Figure 4A), whereas the levels of GSK3β did not differ significantly between the groups (Figure 4B). In the PFC of patients with schizophrenia, there was no significant correlation between the PIK4CA expression level and chlorpromazine equivalent dose (CPZeq; Table 1); the Akt level in PFC was also not correlated with CPZeq.

### 2.2. Analysis of mRNA/Protein Expression Correlation of PIK4CA and Akt1

**Figure 5:**
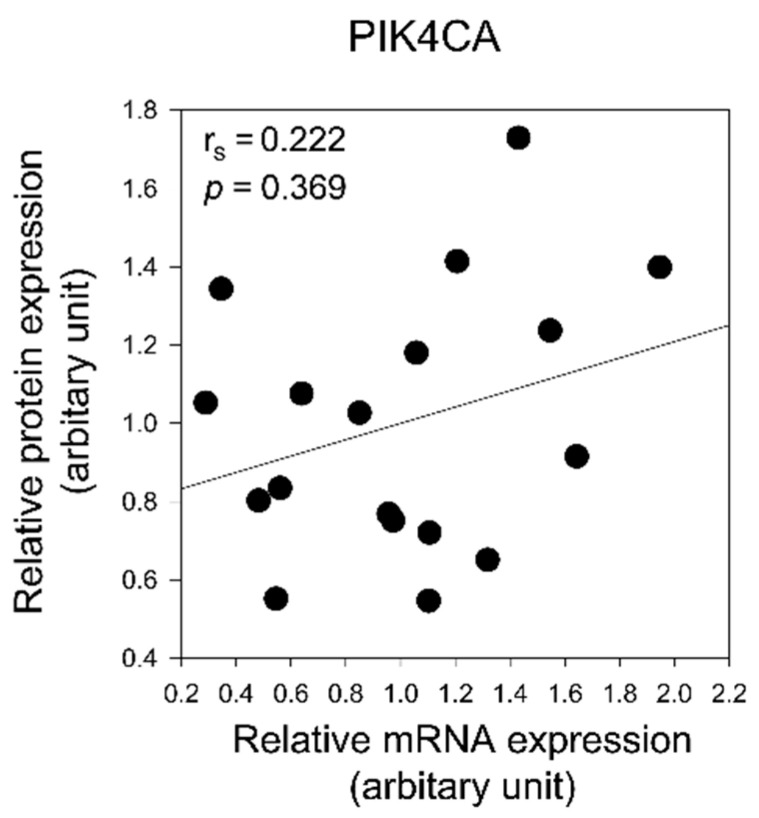
Correlation analysis of mRNA and protein expression of PIK4CA in the prefrontal cortex of patients with schizophrenia. Spearman’s correlation coefficient (r_s_) and *p* value are indicated. The lines indicate the correlation trend.

**Figure 6:**
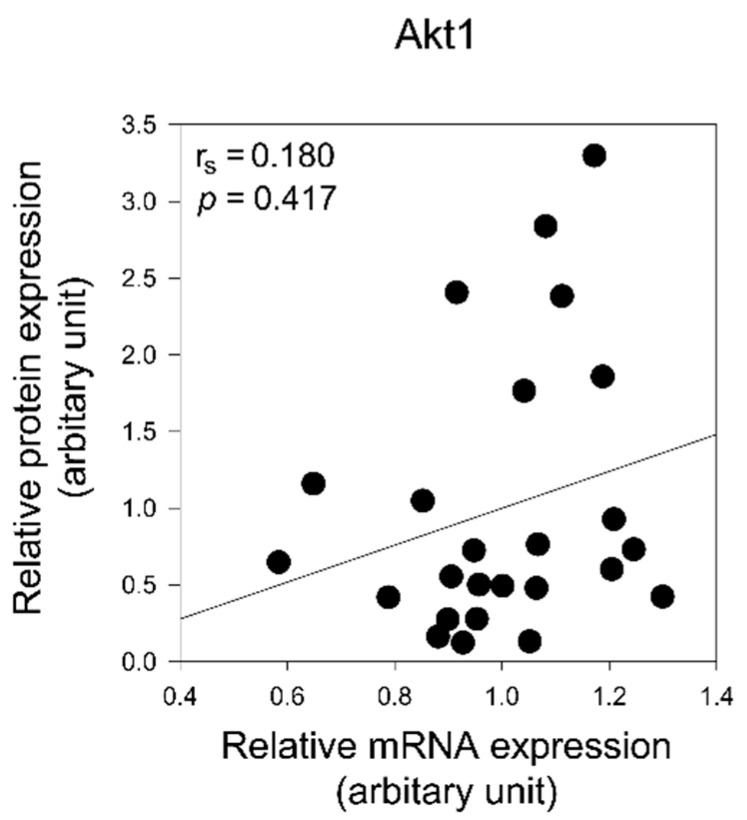
Correlation analysis of mRNA and protein expression of Akt1 in the prefrontal cortex of patients with schizophrenia. Spearman’s correlation coefficient (r_s_) and *p* value are indicated. The lines indicate the correlation trend.

To confirm whether the expression levels of PIK4CA and Akt1 proteins are regulated by mRNA expression levels, we analyzed the correlation between mRNA and protein expression of PIK4CA and Akt1. There was no significant correlation between mRNA and protein expression for either PIK4CA or Akt1 (Figure 5 and Figure 6).

### 2.3. Effects of Phospholipid Signaling-Associated Molecule Genotype on Their Protein Expression

**Figure 7:**
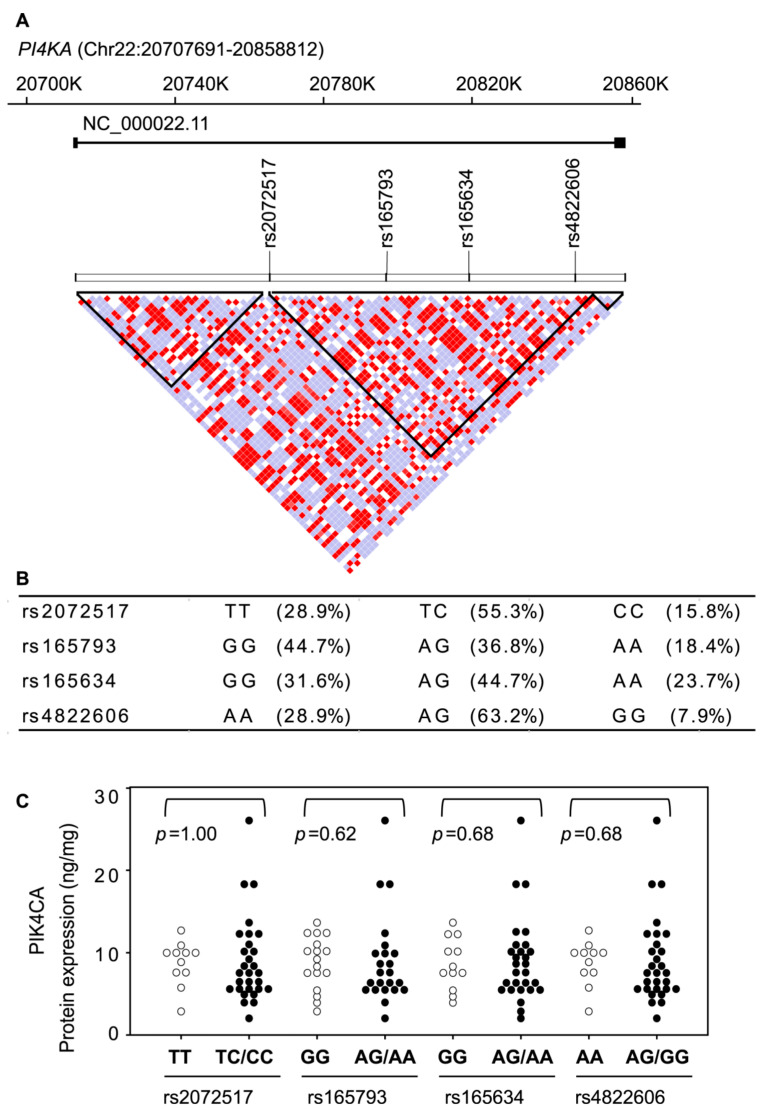
(**A**). Linkage disequilibrium map of *PI4KA* locus, the gene encoding PIK4CA protein showing the four SNPs (rs165634, rs165793, rs2072517, and rs4822606) analyzed in this study. For linkage disequilibrium analysis, we used the Japanese healthy controls in the database of the 1000 Genomes Project. Haploblocks were drawn using Haploview v.4.2 software with standard color scheme. (**B**). The allele frequencies of each SNPs of *PI4KA*. (**C**). The effect of SNPs variants of *PI4KA* on the protein expression of PIK4CA. The expression levels of proteins were compared between groups using the Mann–Whitney *U*-test.

We focused on SNPs of *PI4KA*, the gene that encodes PIK4CA, and compared the protein expression levels with the genotypes of these SNPs. Among 32 SNPs of *PI4KA* included in the chip, four (rs165634, rs165793, rs2072517, and rs4822606) were retrieved for the analysis based on the criteria described in Section 4. We did not identify any SNPs that significantly affected the expression of proteins investigated in this study (Figure 7).

## 3. Discussion

Our study is the first analysis of the expression levels of enzymes associated with the metabolic pathway of PIs in the postmortem brains of patients with schizophrenia. We showed that the expression levels of PIK4CA were significantly lower in the PFC of patients with schizophrenia than in controls. Among the molecules examined in this study, the protein expression of PIK4CA, located upstream of the metabolic pathway of PIs, was altered in patients with schizophrenia, but there was no change in the expression of molecules located downstream. Additionally, among molecules related to the signaling pathway of PIs, the expression of Akt was significantly higher in patients with schizophrenia than in controls, but the expression of GSK3β was unchanged. We also examined the association between the expression of PIK4CA and its 32 SNPs in postmortem brain samples but did not identify any SNPs that significantly affected protein expression.

PIs affect the regulation of the actin cytoskeleton and contribute to the formation of dendritic spines and development of synapses in nerve tissue [27]. As disturbances during neurodevelopment are implicated in the etiology of schizophrenia, it is supposed that PIs are involved in its pathophysiology. However, it is assumed that there are more than 240 molecular species of PIs in vivo as there are at least 30 combinations of fatty acid residues and eight phosphorylation states [7,8] (Figure 1A). Therefore, it was thought necessary to first focus on the processes related to phosphorylation in order to obtain a complete picture of this pathway. Then, in this study, we investigated the expression of PIP5K1C, PIK4CA, and PTEN, which are enzymes involved in the metabolic pathway of PIs. In detail, PIK4CA phosphorylates PI to produce phosphatidylinositol 4-phosphate (PI(4)P), a phosphatidylinositol 1 phosphate (PIP1), and PIP5K1C phosphorylates PI(4)P to produce phosphatidylinositol 4,5-bisphosphate (PI(4,5)P2), a PIP2; meanwhile, PTEN dephosphorylates PIP3 to produce PI(4,5)P2 (Figure 1B). In this way, the molecules analyzed in this study are involved upstream, midstream, and downstream of the metabolic pathway of PIs, respectively [7,28]. Our results indicate that further detailed analyses should focus on the molecules upstream of this complex lipid metabolic pathway.

Considering their role as precursors of two critical second messengers, inositol trisphosphate (IP3(1,4,5)) and diacylglycerol (DAG), PIs are pivotal phospholipids [7,29] (Figure 1B). IP3(1,4,5) and DAGs are produced from PI(4,5)P2 by phospholipase C (PLC). PLCβ-1-knockout mice exhibit a schizophrenia-like phenotype, with an increased incidence of adult hippocampal neurogenesis [30], and deletions of PLCβ-1 have been observed in the orbitofrontal cortex of patients with schizophrenia [31]. Therefore, we assume that the lower PIK4CA level located upstream of this pathway might be related to the pathogenesis of schizophrenia through decreased DAG or IP3(1,4,5)-mediated signaling activity. Moreover, PI(4,5)P2, produced by the enzymatic function of PIK4CA, interacts with proteins involved in membrane transport. In detail, PI(4,5)P2 plays a pivotal role in membrane trafficking and the regulation of synaptic and dense core vesicle exocytosis, namely glutamate and dopamine release [28], which are the main etiological neurotransmitters of schizophrenia. Interestingly, lithium carbonate, which has been used to augment antipsychotics in the treatment of schizophrenia [32], affects the synthesis of PIP2 and subsequent generation of IP3(1,4,5) and DAG [33]. Taken together, decreased PIK4CA levels might be associated with the etiology of schizophrenia, and this protein seems to be a promising therapeutic target.

The locus of *PI4KA* is located on chromosome 22q11, which has been suggested to be strongly associated with schizophrenia. In a Dutch genetic association study of 310 cases and 880 controls, association analysis of 138 myelin-related genes using 771 SNPs demonstrated that SNPs of *PI4KA* are the SNPs most significantly associated with schizophrenia [34]. This result, that *PI4KA* SNPs are associated with schizophrenia, has been reconfirmed in several studies [35,36], but not in Japan [37]. Among the *PI4KA* SNPs associated with schizophrenia, only one, rs165793, was included in our current study, but it did not have a significant effect on PIK4CA protein expression in the postmortem brain.

Additionally, in this study, we elucidated that the protein expression of Akt was significantly higher in the postmortem PFC of patients with schizophrenia than in that of controls. Several previous studies have shown that the levels of Akt protein and phosphorylated Akt are decreased significantly in postmortem brain tissue of patients with schizophrenia [38,39]. However, one study reported that the phosphorylated/total Akt ratio does not differ between patients with schizophrenia and controls [40]; we also observed this in our previous study, which showed that the level of phosphorylated Akt was increased in the PFC of patients with schizophrenia [41]. However, there were many reports that the protein expression level of Akt decreased in the postmortem brain with schizophrenia, including a very recent article [42], which was not consistent with the results of our study. On the other hand, we have found that there are gene polymorphisms that have opposite effects on molecular expression in the brain between Japanese and Caucasians [43], and it is possible that opposite patterns of molecular expression in the brain appear depending on race or ethnicity. In any case, the protein expression results obtained in this study need to be validated using pairwise matched cohorts and by other methods such as Western blotting. While our results were not in accordance with the published literature, it seems that Akt expression was altered in the brain of patients with schizophrenia. Moreover, as PI(3,4,5)P3 activates Akt by recruiting it to the plasma membrane, it is possible that lower PIK4CA levels impact Akt signaling, and dysfunction of this pathway may be a mechanism of schizophrenia pathology.

Meanwhile, we did not show significant correlation between mRNA and protein expression for either PIK4CA or Akt1. Postmortem analyses of the brains of patients with schizophrenia have extensively investigated the expression levels of mRNA. Even though alterations in mRNA expression likely relate to certain biological phenomena or disease, changes in mRNA levels do not always reflect those in protein levels, which in turn directly determine physiological activities. From the point of view of drug discovery, elucidating levels of protein expression is important for choosing novel molecular targets in drug development. Additionally, it would be very interesting to examine what happens by activation of the proteins measured in this study and so this will be considered a top priority for the next target of our research.

This study, conducted using brain tissues, has some limitations that need consideration. First, disease-related confounding factors, including drugs administered antemortem, may have affected protein expression. Although we did not observe any effects of clinical factors, including duration of illness (DOI) and daily dosage of antipsychotic or anticholinergic drugs, on the levels of protein expression, additional animal studies are required to examine the effects of these factors on protein expression in the postmortem brain. Second, our postmortem sample size was relatively small, particularly for a genetic association study. Therefore, the results of this study have to be confirmed by a postmortem examination of a larger cohort. Lastly, since the subjects diagnosed with schizophrenia studied here were not pair matched with comparison subjects by sex, age, and PMI, the results of the statistical analysis should be treated with caution. In the next phase, we need to validate the results in this study with a pairwise matched cohort.

## 4. Materials and Methods

### 4.1. Human Postmortem Brain Tissue

Postmortem brain tissue samples from patients with schizophrenia and control subjects were obtained from Fukushima Brain Bank at the Department of Neuropsychiatry, Fukushima Medical University; Brain Research Institute, Niigata University; and Choju Medical Institute Fukushimura Hospital, Toyohashi as described previously [41]. The use of postmortem human brain tissues was approved by the ethics committees of Fukushima Medical University, Niigata University, and Fukushimura Hospital, and complied with the Declaration of Helsinki (revision in 2013) and its later amendments. All procedures were carried out with the informed written consent of the next of kin. Detailed demographic information of brain tissues from the 23 subjects with schizophrenia and 47 control subjects used in this study is summarized in Table 2. The patients with schizophrenia fulfilled the diagnostic criteria established by the American Psychiatric Association (Diagnostic and Statistical Manual of Mental Disorders: DSM-IV). For patients with schizophrenia, the daily dose of antipsychotics prescribed during the 3 months immediately preceding death is shown as the CPZeq (mg/day; Table 2).

### 4.2. Protein Expression Analysis by Enzyme-Linked Immunosorbent Assay (ELISA) and Multiplex Assay

Pieces of gray matter tissue (weighing approximately 100 mg) from Brodmann area 10 in the PFC were isolated from frozen brains. These frozen brain tissues were suspended in 100 μL of 2% sodium dodecyl sulfate (SDS) solution, incubated for 20 min at room temperature (approximately 20 °C), subjected to three cycles of freeze on dry ice and thaw in water bath, and sonicated for 10 min. Then, the samples were diluted in phosphate buffered saline (137 mM NaCl, 2.7 mM KCl, 10 mM Na_2_HPO_4_, and 1.76 mM KH_2_PO_4_) to ensure that the final concentration of SDS was below 0.2%. After centrifugation (10,000× *g* for 3 min at 4 °C), total protein concentration in the supernatant was measured using the Bradford method (Bradford protein assay kit, Bio-Rad Laboratories, Hercules, CA, USA) with bovine serum albumin as the standard. The expression of proteins was determined by using commercial ELISA kits (SEG843Hu, Cloud Clone Corp, Houston, TX, USA for PIK4CA and MBS282297, MyBioSource, San Diego, CA, USA for PIP5K1C) and multiplex fluorescent bead-based immunoassay kits (MAPmate^TM^ 46-678MAG for PTEN, 46-675MAG for Akt/PKB, and 46-689MAG for GSK3β; Merck Millipore, Tokyo, Japan). The analysis was performed according to the manufacturer’s protocols. The expression levels of each protein were normalized against the total protein concentration.

### 4.3. DNA Collection and SNP Genotyping

Genomic DNA was extracted from the frozen cerebellum or occipital cortex and genotyping was performed using HumanCoreExome -24 v1.0 Beadchip on an iScan system (Illumina, Tokyo, Japan) as described previously [39]. For association analysis between SNPs and protein expression, we excluded SNPs with call rates < 99%, minor allele frequencies < 5%, and Hardy–Weinberg equilibrium test *p*-values < 0.05.

In order to examine the relationship between the selected SNPs, Linkage disequilibrium analysis using Japanese healthy controls in the 1000 Genomes Project database (https://www.internationalgenome.org/, accessed on 29 April 2021) was performed. Haploview v.4.2 software (https://www.broadinstitute.org/haploview, accessed on 29 April 2021) was used for this analysis.

### 4.4. RNA Collection and mRNA Sequencing

Total RNA was isolated from PFC of frozen brain using AllPrep DNA/RNA Mini Kit (Quiagen, Tokyo, Japan). RNA purity was evaluated by the RNA integrity number (RIN) determined using the Agilent 2200 TapeStation (Agilent, Santa Clara, CA, USA). The polyA fraction was isolated from total RNA, followed by its fragmentation. Then double-stranded (ds) cDNA was reverse transcribed from fragmented mRNA. The ds cDNA fragments were processed for adaptor ligation, size selection (for 200 bp inserts) and amplification to generate cDNA libraries. Prepared libraries were subjected to paired-end 2 × 101 bp sequencing on the HiSeq 4000 platform, using HiSeq 3000/4000 SBS Kit.

### 4.5. Statistical Analysis

Demographic variables (sex, age, and postmortem interval) were compared between groups using the χ^2^-test and Student’s and Welch’s *t*-tests. The expression levels of proteins were compared between groups using the Mann–Whitney *U*-test. We also performed Spearman’s rank correlation analysis to investigate the correlation between parameters, namely DOI and CPZeq, and protein expression levels. We also conducted Spearman’s rank correlation test for correlation analysis of mRNA and protein expression. To study the association between SNPs and protein expression, we divided all samples into minor allele carriers and non-carriers for each SNP. The Mann–Whitney *U*-test was used to compare the levels of protein expression between the genotypes of each SNPs. For all test, *p* < 0.05 was considered significant. SPSS ver. 25.0 (SPSS, Chicago, IL, USA) and SigmaPlot ver. 14.0 (Systat Software Inc., San Jose, CA, USA) were used for all analyses.

## 5. Conclusions

In conclusion, our results show that the expression of PIK4CA, located upstream of the metabolic pathway of PIs, was lower in the postmortem PFC of patients with schizophrenia than in that of control subjects, which was accompanied by altered Akt expression in the signaling pathway. Our results reflect the potential molecular mechanisms underlying the pathophysiology of schizophrenia and may result in the development of a novel therapeutic agent.

## Figures and Tables

**Table 1:** Correlations between potential confounding factors (DOI and CPZeq) and protein expression levels.

	DOI	CPZeq
	Spearman’s Rank Test	Spearman’s Rank Test
PIK4CA (μg/mg)	r_s_ = −0.18 (*p* = 0.47)	r_s_ = 0.13 (*p* = 0.57)
PIP5K1C (μg/mg)	r_s_ = −0.28 (*p* = 0.25)	r_s_ = −0.11 (*p* = 0.65)
PTEN (μg/mg)	r_s_ = −0.11 (*p* = 0.66)	r_s_ = 0.32 (*p* = 0.19)
Akt (μg/mg)	r_s_ = −0.14 (*p* = 0.58)	r_s_ = 0.26 (*p* = 0.28)
GSK3β (μg/mg)	r_s_ = −0.08 (*p* = 0.75)	r_s_ = 0.23 (*p* = 0.35)

Spearman’s rank correlation coefficients (r_s_) and *p*-values are listed. DOI: duration of illness, CPZeq: chlorpromazine equivalent dose.

**Table 2:** Demographic information and clinical characteristics of patients with schizophrenia and matched controls.

Variables	Controls	Schizophrenia	*p*-Value
Number of samples	47	23	
Gender			
Female	21	9	0.28 ^b^
Male	26	14	
Race			
Asian	47 (100%)	23 (100%)	
Age at death ^a^ (years)	75.5 (SD 15.8)	69.2 (SD 10.7)	0.05 ^d^
PMI ^a^ (hour)	12.0 (SD 16.3)	16.8 (SD 11.9)	0.21 ^c^
DOI (years)		40.4 (SD 15.0)	
CPZeq (mg/day)		528.3 (SD 647.3)	

PMI: postmortem interval, DOI: duration of illness, CPZeq: chlorpromazine equivalent dose, SD: standard deviation; ^a^ Data are reported as mean ± standard deviation; ^b^ χ^2^-test; ^c^ Student’s *t*-test; ^d^ Welch’s *t*-test.

## Data Availability

The data presented in this study are available on request from the corresponding author. The data are not publicly available due to privacy.
